# A Survey of Standard Protocols for Endodontic Treatment in North of KSA

**DOI:** 10.1155/2014/865780

**Published:** 2014-05-04

**Authors:** Azhar Iqbal, Iftikhar Akbar, Beenish Qureshi, Mohd G. Sghaireen, Mahmoud K. AL-Omiri

**Affiliations:** ^1^Department of Conservative Dentistry, Faculty of Dentistry, AlJouf University, P.O. Box 2014, Sakaka, AlJouf, Saudi Arabia; ^2^Department of Operative Dentistry, Islamabad Medical and Dental College, Bahria University, Islamabad, Pakistan; ^3^Department of Prosthodontics, Faculty of Dentistry, AlJouf University, Sakaka, Saudi Arabia; ^4^Faculty of Dentistry, University of Jordan, Amman, Jordan; ^5^AlJouf University, Sakaka, Saudi Arabia

## Abstract

The objective of this study was to collect information regarding methods, materials, and attitudes employed during the endodontic treatment by dentists in north of Saudi Arabia. A questionnaire was designed and distributed among 300 dentists in north of Saudi Arabia to collect the data about the standard protocols of endodontic treatment. The collected data was analyzed by using the SPSS 10 computer software. Out of a total of 300 surveyed dentists, the 66% response rate showed that this study was true representation of the endodontic treatment performed by the dentists in north of Saudi Arabia. 152 (76%) were general dentists and 48 (24%) were endodontists. 18 (9%) were using rubber dam as the method of isolation during endodontic treatment. 173 (86.5%) were using only measurement radiographs for working length determination and 27 (13.5%) were using both electronic apex locator and measurement radiographs. 95 (47.5%) of the respondents were using standardized technique and 25 (12.5%) were using step-down as a root canal preparation technique. 127 (63.5%) of the respondents were using lateral condensation technique, with gutta percha points for root canal obturation.

## 1. Introduction

The contemporary endodontics involves the introduction of many new instruments, materials, and techniques. Controlled studies have shown that root canal treatment brought high success rates of more than 90% [1]. However, most of these studies have reported data from specialists and university clinics. Therefore, these data do not determine the success rate of endodontic treatment accurately in general dental practice. The success rate of endodontic treatment in general dental practice approximates to 65–75% only [2]. This discrepancy in success rate may reflect a difference in the technical quality of endodontic treatment performed. The quality of endodontic treatment is very important. Therefore, there has to be an entity, the quality of which is being discussed. This entity is standard protocols of endodontic treatment, which are implemented in university study programs and their realization is supervised by trained specialists or experienced general practitioners [3]. After graduation, dentists work independently in unsupervised dental practices where attitude towards existing treatment standards differs. This difference in attitudes towards endodontic treatment performed can lead to the errors that impede the healing process [4]. This difference of attitudes towards endodontic treatment can also make it impossible to accomplish the endodontic treatment according to aseptic principles that are essential for the success of endodontic treatment [4]. Various studies [1–5] have been done to evaluate the success and failure of endodontic treatment, and these studies have shown that the failure could be significantly higher for those teeth which are treated by general dentists not by endodontists [5]. Undergraduate curriculum guidelines have been formulated by the European Society of Endodontology to define the acceptable standard of care in clinical endodontics [6]. Several studies, however, have reported that the majority of dentists are not in compliance with these guidelines [7, 8]. These studies have investigated the attitudes of dentists in western countries, such as Denmark [8], UK [9], Belgium [7], and USA [9]. Other studies have investigated the attitude of general dental practitioners towards the various aspects of endodontic treatment in developing countries [10, 11]. Very few studies have investigated the attitudes of dentists towards endodontic treatment in Saudi Arabia. The aim of this study was to collect information regarding knowledge, materials, methods, and attitudes employed during endodontic treatment by dentists in KSA to evaluate and improve the quality of practice of endodontic treatment in KSA.

## 2. Materials and Methods

A self-administered questionnaire was designed containing the information about methods of isolation, methods of working length determination, techniques of root canal preparation, root canal irrigants, intracanal medicaments, and methods of root canal obturation. The prepared questionnaire was piloted and distributed among 300 dentists in north of KSA and informations about the standard protocols of root canal treatment were collected. The collected data was analyzed by using SPSS 10 computer software to get the results. Simple descriptive analysis was used to get the results as frequencies and percentages.

## 3. Results

Out of the total 200 respondents, 152 (76%) were general dentists and 48 (24%) were endodontists. 102 (51%) of the total respondents were always taking preoperative radiographs during the root canal treatment. Majority of the respondents, 133 (66.5%) stated that they performed the root canal treatment of molar teeth in more than three visits followed by 55 (27.5%) performed in three visits and 12 (6%) performed in one visit.

## 4. Discussion

The response rate of 66% in the present study is the true representation of the standard protocols of endodontic treatment followed by the dentists in north of KSA. The obtained results are reliable evaluation of the standard protocols of endodontic treatment in north of KSA [12]. This study showed that the percentage of general dental practitioners, who were performing the root canal treatment, was high, that is, 76%, when compared with some developing countries like Kenya where this percentage is only 63% [13] and this percentage was low when compared with some developed countries like USA where it was 89% [14]. The present study showed that only 6% of GDPs were completing the root canal treatment in one visit and majority of them, that is, 94%, were completing the procedure in three or more than three visits, whereas in USA, 34.7% of dentists were completing the root canal treatment in one visit for the teeth with normal periapex and 16.2% of the dentists were doing so in the teeth with apical periodontitis [15]. All of the endodontic procedures should be carried out with the application of rubber dam and it should be considered as a standard of care [16]. Despite the importance of rubber dam application, only 9% (Table 1) of the total responding dentists in the present study were using rubber dam and majority of the respondents, that is, 91% (Table 1), were using cotton rolls for isolation during endodontic treatment. In UK [17], only about a quarter of respondents routinely used rubber dam during root canal therapy; however, in America 59% [18] and in New Zealand 57% [19] of the dentists were using rubber dam routinely in endodontic treatment. In a similar study, conducted in KSA, only 3% of the respondents were using rubber dam as the method of isolation. This lack of use of rubber dam can directly affect the standard of root canal treatment and decrease the success rate [20]. The determination of working length is the most crucial step in the endodontic treatment [21]. In the present study, 86.5% of the dentists were using measurement radiographs only to determine the working length. However, radiographic method has inherent inaccuracies, as the apical foramen is not detectable on radiograph [22]. Electronic apex locator has the advantage of being able to locate the apical foramen [23, 24]. Therefore, it is very logical to combine the use of electron apex locator and radiographs to make an efficient and accurate determination of working length. In the present study, only 13.5% of the respondents were using this combination for working length determination. In another study conducted in KSA, a majority of the general dental practitioners were using radiographs and tactile sensation to determine the working length and only 7% of them were using electron apex locators to determine the working length [25]. However, the use of tactile sensation to determine the working length cannot be recommended, because the instrument may bind against the canal wall at any position [26]. The sodium hypochlorite is recommended as the material of choice for irrigating the root canal system because of its effective antimicrobial and tissue dissolving action [27, 28]. In the present study, 31% (Table 2) of the surveyed dentists were using sodium hypochlorite as the root canal irrigant. However a study conducted in Hong Kong showed that the sodium hypochlorite seemed to be gaining popularity 18 because sodium hypochlorite was recommended by the dental schools in Hong Kong. In a study of Whitten et al. [18], 79% of the general dental practitioners used sodium hypochlorite as irrigant, while in a survey of Whitworth et al. [17] in UK, the local anesthetic solution was the most commonly used irrigant for endodontic treatment. Possibly the limited use of rubber dam was a factor in the choice of root canal irrigant. The main objective of the use of intracanal medicaments is to reduce the number of bacteria, to relieve pain, to reduce inflammation, and to dry the wet canals [29]. The present study showed that majority of the dentists, that is, 55% (Table 2), were using formocresol as intracanal medicament. Formocresol has many adverse effects due to its mutagenicity [30]. Although calcium hydroxide is considered as standard intracanal dressing, only 15% (Table 2) of the dentists in the present study were using this. The general dental practitioners must be encouraged to use it in place of formocresol since it has multiple biological functions [31, 32]. Majority of the dentists 82.5% (Table 2) in the present study were using stainless steel hand instrument for root canal preparation and only a very small percentage of the dentists (17.5%) (Table 2) were using nickel titanium rotary and hand instruments, indicating that new developments are being slowly and gradually adopted by the dentists in KSA. The standardized technique of root canal preparation has some disadvantages like overpreparation resulting in elliptically shaped defect at the end-point of preparation [33], which could result in incomplete obturation of the root canal system. Therefore those preparation techniques which involve the early coronal flaring, for example, crown-down technique, should be adopted as they will produce a better shape and enhanced penetration of irrigating solution [34]. But unfortunately in present study, only 12.5% (Table 1) of the dentists were using this technique and 47.5% (Table 1) were using standardized technique [34]. Root canal obturation prevents the ingress of microorganisms into already cleaned root canal system. Lateral condensation of gutta percha with sealer is a relatively simple, versatile, universally acknowledged, and most common obturation technique that has produced good results and does not require expensive equipment [35, 36]. In the present study, 63.5% (Table 1) of the respondents were using lateral condensation technique with a root canal sealer as the root canal obturation technique. In a similar study, the lateral condensation technique with sealer was the most common, that is, 87%, among the surveyed general dental practitioners [35]. Similarly almost half of the general dental practitioners in north Jordan used lateral condensation of gutta percha to obdurate the root canal space. A similar study conducted in Saudi Arabia showed that 65% of the general dental practitioners used lateral condensation as an obturation technique. The present study showed that only a very small percentage, that is, 1.5% (Table 1), of the general dental practitioners were using modern obturating techniques like injectable obturating technique and thermafil.

## 5. Conclusions

Considerable advances have been made in materials and techniques over the last decade in the endodontics, which made it highly dynamic and evolving discipline of dentistry. There are a limited number of endodontic specialists in KSA. Therefore, the professional bodies should promote the endodontic specialty programmes to increase the number of endodontic specialists in the kingdom. There should also be conduction of properly structured continuing education courses in endodontics, under the supervision of highly professional endodontic specialists, to meet the demands and needs of general dental practitioners regarding the modern equipment and new techniques in endodontics.

## Figures and Tables

**Table 1:** The techniques used for root canal isolation, preparation, and obturation.

Technique	Frequency (*n*)	Percentage (%)
Root canal isolation		
Rubber dam	18	9
Cotton rolls	181	91
Root canal preparation		
Standardized technique	95	47.5
Step-back technique	82	41
Crown-down technique	25	12.5
Root canal obturation		
Lateral condensation	162	81
Single cone	25	12.5
Paste filling	10	5
Injectable and thermafil	3	1.5

**Table 2:** The frequency of standard chemicals and instruments for endodontic treatment.

Chemicals and instruments	Frequency (*n*)	Percentage (%)
Types of root canal irrigants		
Sodium hypochloride	62	31
Saline	110	55
Hydrogen peroxide	28	14
Intracanal medicaments		
Calcium hydroxide	10	5
Camphorated monochlorophenol	50	25
Formocresol	110	55
Instruments used for root canal preparation		
Stainless steel hand instruments (K-File, H-File, and Reamer)	165	82.5
Ni-Ti hand and rotary instruments	35	17.5

## References

[1] Sjögren U, Hägglund B, Sundqvist G, Wing K (1990). Factors affecting the long-term results of endodontic treatment. *Journal of Endodontics*.

[2] Eriksen HM (1991). Endodontology—epidemiologic considerations. *Endodontics & dental traumatology*.

[3] European Society of Endodontology (2002). Consensus report of the European Society of Endodontology on quality guidelines for endodontic treatment. *International Endodontic Journal*.

[4] Gorni FGM, Gagliani MM (2004). The outcome of endodontic retreatment: a 2-yr follow-up. *Journal of Endodontics*.

[5] Weiger R, Axmann-Krcmar D, Löst C, Weiger R (1998). Prognosis of conventional root canal treatment reconsidered. *Endodontics and Dental Traumatology*.

[6] European Society of Endodontology (2001). Undergraduate curriculum guidelines for endodontology. *International Endodontic Journal*.

[7] Jenkins SM, Hayes SJ, Dummer PMH (2001). A study of endodontic treatment carried out in dental practice within the UK. *International Endodontic Journal*.

[8] Slaus G, Bottenberg P (2002). A survey of endodontic practice amongst Flemish dentists. *International Endodontic Journal*.

[9] Whitten BH, Gardiner DL, Jeansonne BG, Lemon RR (1996). Current trends in endodontic treatment: report of a national survey. *Journal of the American Dental Association*.

[10] Akpata ES (1984). Endodontic treatment in Nigeria. *International Endodontic Journal*.

[11] Al-Omari WM (2004). Survey of attitudes, materials and methods employed in endodontic treatment by general dental practitioners in North Jordan. *BMC Oral Health*.

[12] Bulman JS, Osborn JF (1989). *Statistics in Dentistry*.

[13] Maina SW, Ng’ang’a PM (1991). Root canal treatment and pulpotomy in Kenya. *East African Medical Journal*.

[14] Wasilkoff PC, Maurice CG (1976). Role of endodontics in current dental practice. *The Journal of the American Dental Association*.

[15] Gatewood RS, Himel VT, Dorn SO (1990). Treatment of the endodontic emergency: a decade later. *Journal of Endodontics*.

[16] Silversin JB, Shafer SM, Sheiham A, Smales FC (1975). The teaching and practice of some clinical aspects of endodontics in Great Britain. *Journal of Dentistry*.

[17] Whitworth JM, Seccombe GV, Shoker K, Steele JG (2000). Use of rubber dam and irrigant selection in UK general dental practice. *International Endodontic Journal*.

[18] Whitten BH, Gardiner DL, Jeansonne BG, Lemon RR (1996). Current trends in endodontic treatment: report of a national survey. *Journal of the American Dental Association*.

[19] Koshy S, Chandler NP (2002). Use of rubber dam and its association with other endodontic procedures in New Zealand. *New Zealand Dental Journal*.

[20] Christensen GJ (1994). Using rubber dams to boost quality, quantity of restorative services. *The Journal of the American Dental Association*.

[21] Ricucci D (1998). Apical limit of root canal instrumentation and obturation, part 1. Literature review. *International Endodontic Journal*.

[22] Olson AK, Goerig AC, Cavataio RE, Luciano J (1991). The ability of the radiograph to determine the location of the apical foramen. *International Endodontic Journal*.

[23] Pagavino G (1998). A SEM study of in vivo accuracy of the root ZX electronic apex locator. *Journal of Endodontics*.

[24] de Moor RJG, Hommez GMG, Martens LC, de Boever JG (1999). Accuracy of four electronic apex locators: an in vitro evaluation. *Endodontics & Dental Traumatology*.

[25] de Moor RJG, Hommez GMG, de Boever JG, Delmé KIM, Martens GEI (2000). Periapical health related to the quality of root canal treatment in a Belgian population. *International Endodontic Journal*.

[26] Dummer PM, McGinn JH, Rees DG (1984). The position and topography of the apical canal constriction and apical foramen. *International Endodontic Journal*.

[27] Bystrom A, Sundqvist G (1983). Bacteriologic evaluation of the effect of 0.5 percent sodium hypochlorite in endodontic therapy. *Oral Surgery Oral Medicine and Oral Pathology*.

[28] Wadachi R, Araki K, Suda H (1998). Effect of calcium hydroxide on the dissolution of soft tissue on the root canal wall. *Journal of Endodontics*.

[29] Bystrom A, Claesson R, Sundqvist G (1985). The antibacterial effect of camphorated paramonochlorophenol, camphorated phenol and calcium hydroxide in the treatment of infected root canals. *Endodontics & Dental Traumatology*.

[30] Block RM, Lewis RD, Hirsch J, Coffey J, Langeland K (1983). Systemic distribution of [^14^C]-labeled paraformaldehyde incorporated within formocresol following pulpotomies in dogs. *Journal of Endodontics*.

[31] Abbott PV (1990). Medicaments: aids to success in endodontics. Part 2. Clinical recommendations. *Australian Dental Journal*.

[32] Foreman PC, Barnes IE (1990). Review of calcium hydroxide. *International Endodontic Journal*.

[33] Weine FS, Kelly RF, Lio PJ (1975). The effect of preparation procedures on original canal shape and on apical foramen shape. *Journal of Endodontics*.

[34] Fava LRG (1983). The double-flared technique: an alternative for biomechanical preparation. *Journal of Endodontics*.

[35] Walton RE, Johnson WT, Walton RE, Torabinejad M (2002). Obturation. *Principles and Practice of Endodontics*.

[36] Qualtrough AJE, Whitworth JM, Dummer PMH (1999). Preclinical endodontology: an international comparison. *International Endodontic Journal*.

